# QuickStats: Percentage of Deaths,* by Place of Death^†^ — National Vital Statistics System, United States, 2000–2018

**DOI:** 10.15585/mmwr.mm6919a4

**Published:** 2020-05-15

**Figure:**
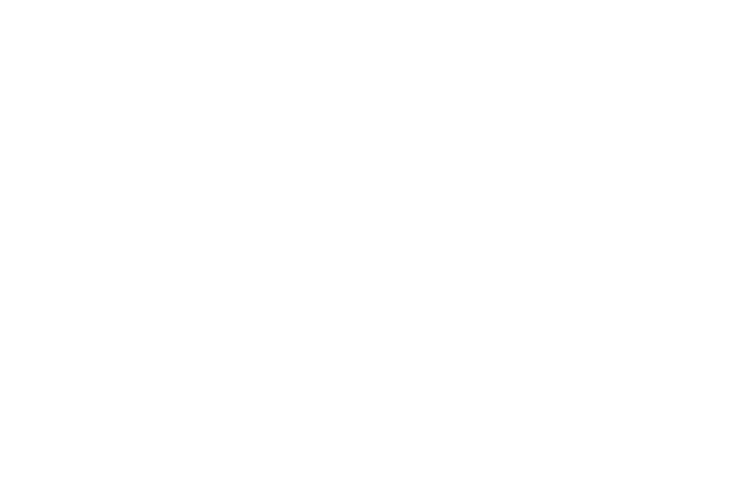
The percentage of deaths from all causes that occurred in a hospital decreased from 48.0% in 2000 to 35.1% in 2018. During that period, the percentage of deaths that occurred in the decedent’s home increased from 22.7% to 31.4%, and the percentage that occurred in a long-term care facility (hospice, nursing home, long-term care) increased from 22.9% to 26.8%.

**Source:** National Vital Statistics System. Underlying cause of death data, 2000–2018. https://wonder.cdc.gov/ucd-icd10.html.

